# Mississippi State Medical Association

**Published:** 1872-05

**Authors:** J. W. M. Shattuck


### MISSISSIPPI STATE MEDICAL ASSOCIATION.

The fifth annual session of the Mississippi State Medical Association was held on the 3rd and 4th of April, 1872, at Holly Springs.

The meeting was called to order by the President, Dr. W. M. Compton, of Jackson, Dr. J. W. M. Shattuck, of Columbus, Secretary.

The session was opened with prayer by Rev. Mr. Craig, of Holly Springs.

The attendance was not so large as was anticipated, owing to professional engagements of members, unfavorable weather, and other causes; although those present manifested an unusual interest in the proceedings.

The transactions of the fourth annual session were read and adopted as published.

An able and interesting address on "Hints to Medical Experts" was delivered by the President. The address was referred, and ordered printed. Reports were received from the following standing committees:

All written reports were referred to the Committee of Publication and ordered printed, either in THE GEORGIA MEDICAL COMPANION and the Louisville Medical *Journal*, or in pamphlet form with the proceedings of the Association.

Report on memorializing the State Legislature in reference to establishing an "Eye and Ear Infirmary," by Dr. Edward Lea, of Durant; on memorializing the Legislature in reference to securing "Physicians' Fees," by Dr. W. M. Compton, of Jackson; "Chloroform in Obstetrics," by Dr. C. B. Galloway, Canton; "Conservative Surgery," by Dr. A. A. Lyon, Columbus; "Hypodermic Medication," by Dr. Shattuck, Columbus; "Pneumonia," by Dr. J. M. Lewis, Kosciusco; "Chronic Constipation," by Dr. J. M. Taylor, Corinth; "Tranchonia and Malaria," Dr. E. Lea, Durant; "New or Improved Surgical Instruments," by Dr. Kline, exhibiting to the members a large number of surgical and obstetrical instruments received from the manufacturer, Mr. Tiemman, of New York.

Volunteer scientific papers were also received: one on "Malarial Hermaturia," from Dr. E. W. Anderson, of Kirkwood, and several members of the Columbus and Lowndes County Medical Society, including a synopsis of the proceedings of that Society for the past year.

These papers, being considered too voluminous to be read in open session, were referred to the Committee on Contributions, and upon their recommendation, they were ordered printed.

Standing committees were appointed to report at the regular meeting in 1874, upon the following topics, viz:

On "Malarial Hemorrhagic Fever," Dr. J. P. Moore, Yazoo City; "Cerebro-Spinal Meningitis," Dr. S. V. D. Hill, Macon;

"Sequelæ of Malarial Diseases," Dr. Shattuck, Columbus; "Foreign Bodies in the Air Passages," Dr. Burche, Yazoo City; "Progress of Materia Medica and Therapeutics," Dr. Boothe; "Epidemic Fever" at Jackson, Vicksburg, and Natchez, in the fall of 1871, Dr. Whitehead, Vicksburg; "Albuminuria in Pregnancy," Dr. Dancy, Holly Springs; "Progress of Surgery," Dr. Gadbsberry, Yazoo City; "Conservative Surgery," Dr. Lyon, Columbus; "Surgical Records," Dr. L. G. Capers, Vicksburg; "Contagious and Epidemic Diseases," Dr. Hicks, Vicksburg; "Improved Treatment of Uterine Diseases," Dr. Kline, Meridian; "Scarlatina and its Sequelæ," Dr. Barnett, Vicksburg; "Dysmenorrhœa," Dr. McConnell, Brownsville; "The Urine in Malarial Diseases," Dr. Sykes, Aberdeen; "Thermometer in Malarial Diseases," Dr. Gholson, Holly Springs.

The Committee on Publication were instructed to publish and distribute a circular to physicians in all parts of the State, urging the formation of local medical societies in their respective sections; also requesting them to forward to the Recording Secretary the names and post-office address of as many regular physicians as they are able to ascertain.

During the session fifteen new members were registered. The Association now numbers eighty-five members, embracing some of the best medical talent in the State.

The following officers were elected for the present year:

Dr. C. B. Galloway, President, Canton.
Dr. D. W. Boothe, First Vice-President, Vicksburg.
Dr. W. M. Lea, Second Vice-President, Holly Springs.
Dr. J. D. Burche, Third Vice-President, Yazoo City.
Dr. Leo. Shackleford, Fourth Vice-President, Meridian.
Dr. J. W. M. Shattuck, Recording Secretary, Columbus.
Dr. P. F. Whitehead, Corresponding Secretary, Vicksburg.
Dr. W. G. Sykes, Treasurer, Aberdeen.
Dr. A. A. Lyon, Orator, Columbus.

Delegates to the American Medical Association, Drs. Hicks, Vicksburg; Gadberry, Yazoo City; Lipscomb, Columbus; Isom, Oxford; Shackleford, Meridian; Compton, Jackson; Taylor, Corinth; and Gholson, Holly Springs.

A committee was appointed to make all necessary arrangements for the next meeting of the Association. It will be held at Vicksburg, on the first Wednesday in April, 1873.

J. W. M. Shattuck, M. D.,
Recording Secretary.

Messrs. Editors.—The Prospects of our State Association are very bright at this time; our President, Dr. C. B. Galloway, of Canton, is a man of ability, energy, and large experience in his profession, and we feel confident that our membership will be largely increased before the expiration of another year.

This can easily be done if the individual members of the profession will exert themselves. Other States are making rapid advances in this particular, and we should not fail to catch this spirit of progress. We have abundant material in the State, and it ought to be brought into service.

We are intending to make greater efforts than ever, this year, to have a large attendance at our next meeting. The habit of attending medical associations is like any other, it must be acquired by experience; if we can get them once or twice they will become regular attendants. Stir them up through your journal—urge the organization of local medical societies. It is very difficult to persuade physicians to perform their duty through and in these societies—they must have "line upon line"—but they make first-rate workers when once enlisted. Say to the Georgia State Medical Asssociation that we intend to outnumber them at our next meeting.

Respectfully,
J. W. M. Shattuck, M. D.

